# Expert opinion of mediastinal lymph node positions from an intrabronchial view

**DOI:** 10.1186/s12890-016-0176-6

**Published:** 2016-01-22

**Authors:** Kjetil Roth, Tomas Eagan, Jon Hardie

**Affiliations:** Department of Internal Medicine, HMR Hospital Trust, 6026 Ålesund, Norway; Department of Clinical Science, Faculty of Medicine and Dentistry, University of Bergen, Bergen, Norway; Department of Thoracic Medicine, Haukeland University Hospital, Bergen, Norway

**Keywords:** Bronchoscopy, Lung Neoplasms, Diagnosis, Lymph nodes, Observer variation

## Abstract

**Background:**

The knowledge of the mediastinal lymph node positions from an intrabronchial view was important for conventional transbronchial needle aspiration (TBNA). The introduction of endobronchial ultrasound guided transbronchial needle aspiration (EBUS-TBNA) changed the focus from the intrabronchial landmarks to the real life ultrasound images. However when all EBUS reachable lymph nodes are evaluated (mapping), the knowledge of the intrabronchial positions is crucial. The objective of this study was to present a new expert opinion map from an intrabronchial perspective validated by an interobserver variation analysis.

**Methods:**

Physicians who had performed more than 30 EBUS-TBNA were included. They marked areas for optimal TBNA sampling on standardized pictures from an intrabronchial perspective. Areas marked by more than 3 of the 14 experts who had performed more than 1000 EBUS provided the data for the map. The map was validated among the experts and the agreement was compared to the agreement among less experienced physicians.

**Results:**

There was high agreement (>80 %) among the experts in lymph node positions 4 L, 7, 10 L, 11R and 11 L. The agreement for 4R and 10R was low (<70 %). The agreement among the most experienced physicians was significantly higher than the less experienced physicians in station 10 L (92 % vs. 50 %, p:0.01).

**Conclusions:**

It was possible to present a new map of expert opinion for optimal sampling positions in lymph node stations 4 L, 4R, 7, 10 L, 11R and 11 L. All positions except 4R had high agreement. No area was covered by more than 3 of the 14 experts in station 10R.

## Background

Non-small cell lung cancer (NSCLC) is currently the cancer with the highest mortality in the Western world [1], where survival still is mostly dependent upon whether the cancer is resectable or not. Vital to the staging and thus determination of resectability is assessment of the hilar and mediastinal lymph nodes. Transbronchial needle aspiration (TBNA) was introduced by Schiepatti in 1949 [2]. Conventional TBNA was performed based on the knowledge of the lymph node positions from an intrabronchial perspective [3] and later guided by the computer tomography (CT) scan for each patient [4]. Endobronchial ultrasound guided transbronchial needle aspiration (EBUS-TBNA) has gradually replaced conventional TBNA. The knowledge of the lymph node positions from an intrabronchial map is not crucial for “hit and run” EBUS, but will be crucial for a systematic mapping of all lymph nodes in the mediastinum [5].

In 1997 Mountain and Dresler [6] updated the American Thoracic Society (ATS) map [7]. The International Association for the Study of Lung Cancer (IASLC) established a Lung Cancer Staging project in 1998 and published a new description in 2009 [8]. Neither Mountain and Dresler nor IASLC provided maps of the lymph node stations from the intrabronchial perspective.

There are some point estimates of the lymph nodes from an intrabronchial perspective, but they do not describe the areas for sampling. Wang published a TBNA map from the intrabronchial perspective based on his own experience with conventional TBNA in 1994 [4]. Wang and Mehta made a new point estimate from the intrabronchial perspective in 2004, based on a reconstruction of CT scans [2]. Finally Ernst and Herth published a similar map in 2009 [9].

The knowledge of the mediastinal lymph node positions from the intrabronchial view must reflect the variation of the lymph node locations. EBUS visualize the exact position of each lymph node. Some physicians have performed more than 1000 EBUS, which represents a great repository of knowledge about the normal variation from the intrabronchial perspective.

The aim of this interobserver study was to present an expert opinion of the areas optimal for lymph node sampling seen from the intrabronchial perspective. The new map was validated by an interobserver analysis.

## Methods

The first author made pictures encompassing positions previously described as optimal for TBNA [2, 9]. A pilot study was conducted, which included four experienced physicians from three centers different than our own. The four physicians were shown the pictures, and subsequently marked the area for their preferred TBNA sampling positions. Based on the pilot study, the pictures were determined to cover the necessary areas for assumed optimal TBNA positions. The first author subsequently contacted all the included experienced physicians in their centers or at conferences. Physicians who had performed at least 30 EBUS-TBNA were eligible for inclusion in the study. The Europe tour (2012) included Krakow, Heidelberg, Leuven and Ancona. Cleveland, Boston, Philadelphia, Baltimore and Atlanta were visited in USA (2013). The other physicians were included at the European respiratory society conference in Vienna (2012) and at a TBNA conference in Lund (Sweden 2012). Out of 48 contacted physicians, 46 were included while two denied to give their opinions.

**Fig. 1:**
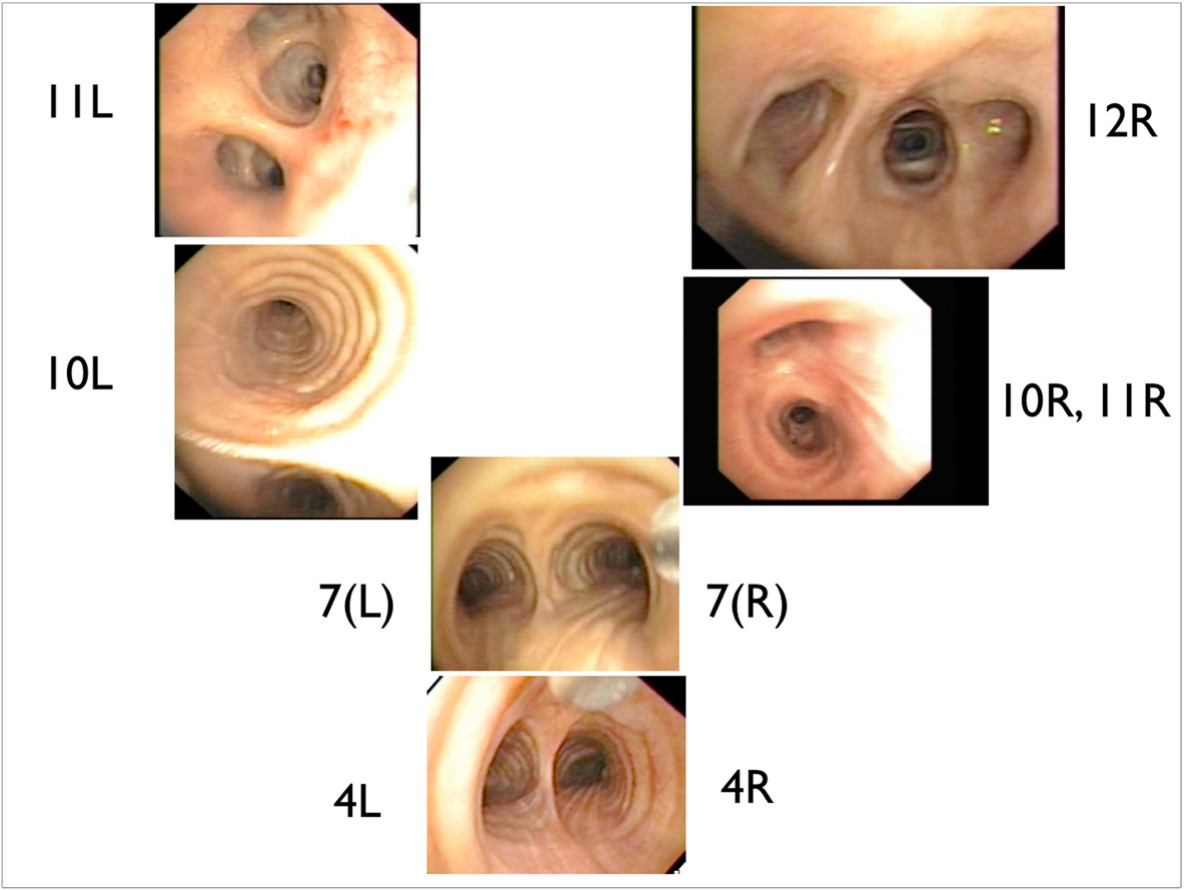
Bronchoscopy pictures of assumed optimal positions for transbronchial needle aspiration (TBNA)

Each physician was shown pictures as shown in Fig. 1. The first author described the different pictures regarding orientation and position of the different bronchial branches. The included physicians were instructed to mark an area, not the exact “pinpoint” position, of assumed optimal TBNA sampling on the different pictures based on the text beside the picture.

All physician-drawings were scanned with ArcSoft photobase [10]. Each drawing was subsequently digitalized based on the scan and the opacity of the drawings was reduced with the program Pixelmator [11]. Finally, each drawing was superimposed with reduced opacity over the original background picture.

**Fig. 2:**
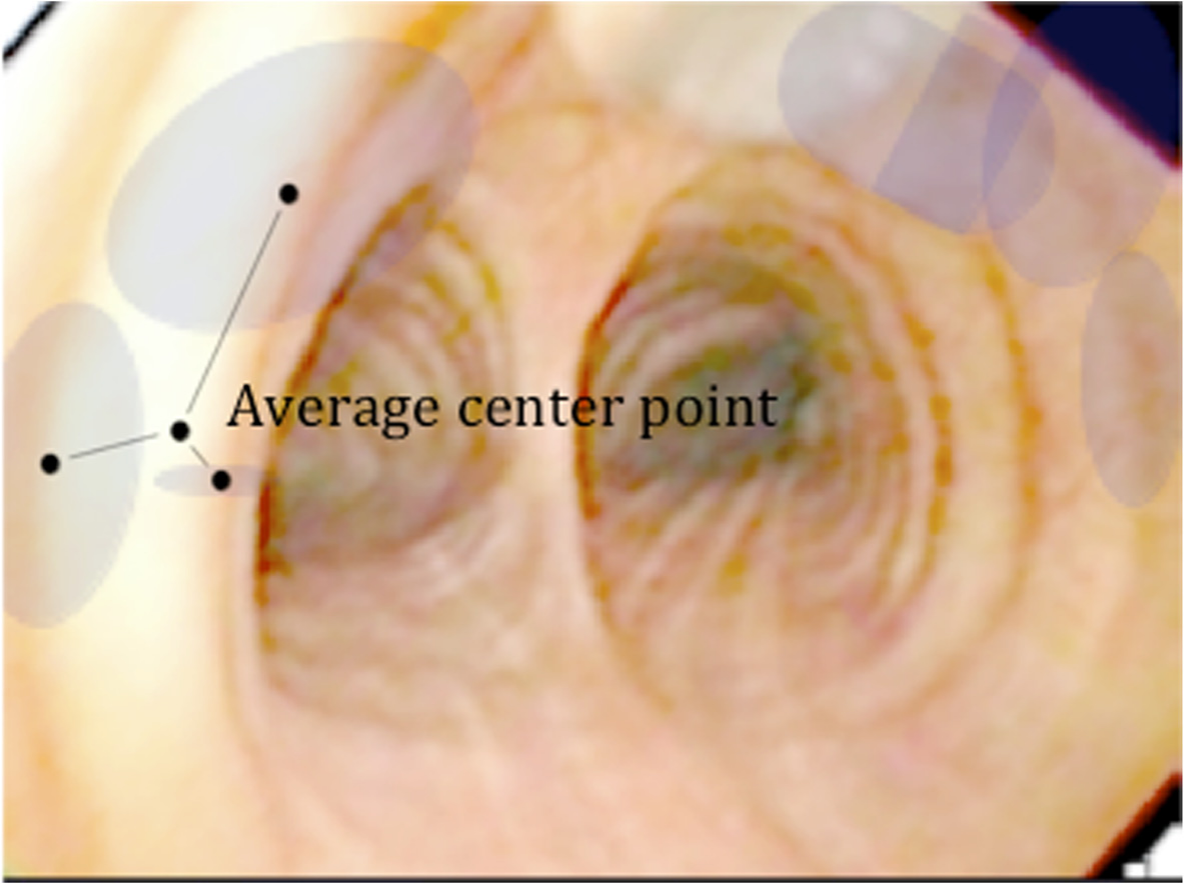
Description of the area and the distance to the average center point. The areas were measured in cm^2^. The distance to the average center point was measured in cm

The areas of each drawing were calculated with Adobe Illustrator [12]. Adobe Illustrator provided the x,y position from the center of the drawings. The average center points and the distances from the average center points were calculated (Fig. 2).

Data from station 12R was excluded because most physicians marked station 12R between the middle lobe and the right lower lobe. This area was previously defined as station 12R by Mountain and Dresler [6], but is now defined as station 11R [8].

The agreement was based on the center points in the drawings. An average center point was calculated from all center points. In addition to agreement for center points, the sizes of the drawings were evaluated. Based on the authors’ clinical experience, a difference in center point of 1 cm will have a clinical impact in the ability to hit the lymph node. Agreement for center point was thus defined as the percentage of physicians with center point within 1 cm from the average center point. The variation in the marked size was evaluated based on the areas. Agreement for area was defined as the percentage of physicians with area less than 2 cm^2^. The median areas drawn by the physicians were below 1 cm^2^ for all lymph node stations except in station 4R. Agreement was divided into three groups: High: >80 % agreement for center point and area, Intermediate: 70–80 % agreement for center point and area, Low: <70 % agreement for center point and area.

The agreement among the most experienced physicians was compared to the agreement among the less experienced physicians. Physicians having performed more than 1000 EBUS-TBNA procedures were classified as experienced.

**Fig. 3:**
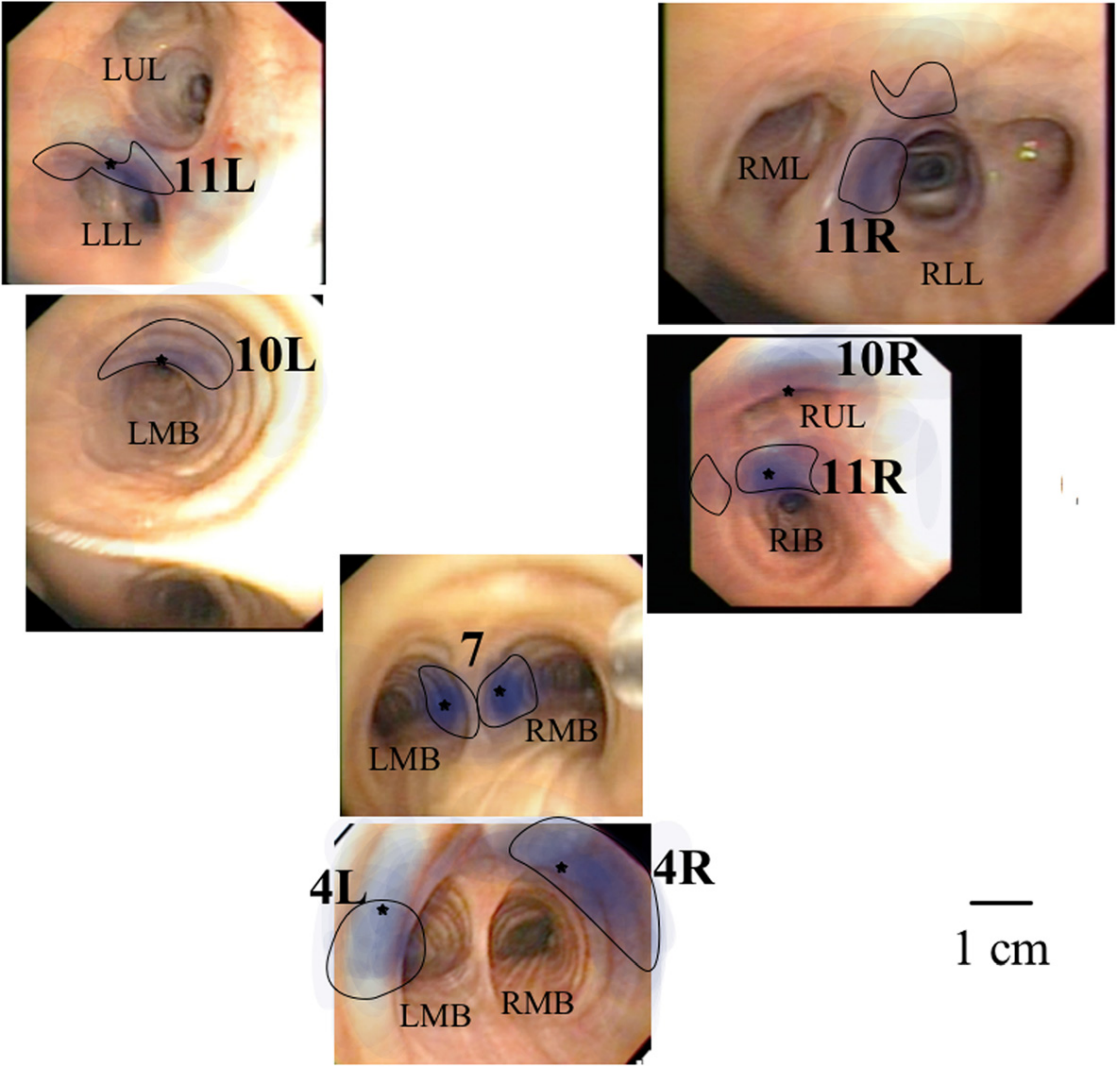
Map of the lymph node stations based on expert opinion. Blue colour: Physician-drawings superimposed over each other with reduced opacity. Star: Average center positions for all physicians. Marked areas: Areas covered by more than 20 % of the 14 physicians that had performed 1000 EBUS or more. LMB: Left main bronchus, RMB: Right main bronchus, RIB: Right intermediate bronchus, RUL: Right upper lobe, RML: Right middle lobe, RLL: Right lower lobe, LLL: Left lower lobe, LUL: Left upper lobe

The new expert opinion map was based on all physician-drawings superimposed over each other with reduced opacity. A star marked the average center points for all physicians. The main borders in the map were areas covered by more than 3 of the 14 physicians who had performed 1000 EBUS or more (Fig. 3).

All statistical analyses were performed with IBM SPSS version 21 [13]. The Norwegian Regional Ethical Committee approved the study. No patient sensitive data were obtained. Each participating specialist was verbally informed of the intentions and procedures of the study and confirmed this with their signing on the diagram and information formulary.

## Results

46 physicians were included between August 2012 and January 2013. The baseline characteristics of the participants in the study are described in Table 1. The EBUS-TBNA volume differed somewhat between hospitals. Approximately 1/3 of the physicians worked in centers with more than 500 EBUS-TBNA procedures yearly, 1/3 worked in hospitals with less than 150 EBUS-TBNA procedures yearly. The most experienced group of physicians (14/46, 30 %) had performed more than 1000 EBUS-TBNA procedures each.

**Table 1:** Baseline characteristics

	n	%
Participants		
Europe	28	60.9
USA	16	34.8
Other	2	4.3
EBUS performed yearly in the hospital		
<150	16	34.8
150–500	12	26.1
>500	16	34.8
EBUS performed ever by participant		
<150	12	27.3
150–499	9	20.5
500–999	9	20.5
>999	14	30.4

The median distances from the average center points and the average areas are described in Table 2 with respective interquartile ranges. There was a close relation between the agreement measured by distances from average center points and the agreement measured by the size of the sampling areas.

**Table 2:** Inter-observer analysis

	Distance from the average center point:		Area:	
	n	Median (IQR)	n	Median (IQR)
Lymph node station:				
*7 (left side)*	33	0.27 cm (0.13-0.38)	33	0,38 cm^2^ (0.27-0.56)
*11R*	43	0.37 cm (0.20-0.65)	43	0.57 cm^2^ (0.36-0.97)
*7 (right side)*	45	0.39 cm (0.23-0.49)	45	0.63 cm^2^ (0.29-0.83)
*11 L*	45	0.55 cm (0.40-0.97)	46	0.75 cm^2^ (0.35-1.11)
*10 L*	40	0.64 cm (0.41-1.06)	40	0.80 cm^2^ (0.37-1.54)
*4 L*	40	0.74 cm (0.44-1.0)	40	0.80 cm^2^ (0.45-1.79)
*4R*	42	0.75 cm (0.50-1.27)	42	1.30 cm^2^ (0.59-2.49)
*10R*	32	0.78 cm (0.44-1.34)	32	0.92 cm^2^ (0.57-1,43)

Q1 = 25 % percentile, Q3 = 75 % percentile. The average center points were calculated as the average of all center points in the drawings for each lymph node station

Table 3 describes the agreement for all participants. The agreement was high (>80 %) for station 11R and 7. There was high agreement for station 7 regardless of sampling from right or left main bronchus. The agreement was intermediate (70–80 %) for 4 L, 10 L, and 11 L, and low (<70 %) for 10R and 4R.

**Table 3:** Agreement for center point and area

	Center point within 1 cm from the average center position		Area < 2 cm^2^		Agreement
	n	%	n	%	
Lymph node station:					
*7 (left side)*	33/33	100 %	33/33	100 %	High
*11R*	41/43	95.3 %	41/43	95.3 %	High
*7 (right side)*	41/45	91.1 %	41/45	91.1 %	High
*11 L*	34/45	75.6 %	42/46	91.3 %	Intermediate
*4 L*	30/40	75.0 %	33/40	82.5 %	Intermediate
*10 L*	29/40	72.5 %	34/40	85.0 %	Intermediate
*10R*	21/32	65.6 %	28/32	87.5 %	Low
*4R*	25/42	59.5 %	30/42	71.4 %	Low

All measurements were based on the drawings. The average center position was the average x coordinate and the average y coordinate of all drawings in the lymph node station. The agreement was defined by the proportion with center point within 1 cm from the average center point, and area below 2 cm^2^; High if >80 %, Intermediate: 70–80 %, Low: <70 %

There was high agreement (>80 %) among the 14 most experienced experts in lymph node positions 4 L, 7, 10 L, 11R and 11 L (Table 4). The less experienced physicians had intermediate or low agreement 4 L, 10 L and 11 L. The difference was significant in 10 L (experts: 12/13, 92 % vs. less experienced: 13/26, 50 %, p = 0.01). Both groups had low agreement for 4R and 10R (ns, Chi square test).

**Table 4:** Comparison of agreement among the most experienced physicians and the other physicians in the study

	>1000 EBUS-TBNA^a^ performed		<1000 EBUS-TBNA^a^ performed		
	Agreement for center point and area		Agreement for center point and area		
	n (%)	Agreement	n (%)	Agreement	p^b^
Lymph node station:					
*7 (left side)*	9/9 (100 %)	High	23/23 (100 %)	High	NS
*11R*	12/13 (92.3 %)	High	26/28 (92.9 %)	High	0.95
*7 (right side)*	12/13 (92.3 %)	High	26/30 (86.7 %)	High	0.60
*11 L*	13/14 (92.9 %)	High	22/30 (73.3 %)	Intermediate	0.14
*4 L*	12/14 (85.7 %)	High	15/24 (62.5 %)	Low	0.13
*10 L*	12/13 (92.3 %)	High	13/26 (50.0 %)	Low	0.01
*10R*	4/8 (50.0 %)	Low	15/23 (65.2 %)	Low	0.45
*4R*	5/12 (41.7 %)	Low	14/28 (50.0 %)	Low	0.63

The agreement was defined by the proportion with distance from average center point below 1 cm and area below 2 cm^2^; High if >80 %, Intermediate: 70–80 %, Low: <70 %. ^a^Endobronchial ultrasound guided transbronchial needle aspiration. ^b^Chi-square test

Figure 3 presents an expert opinion map of the lymph node sampling positions from an intrabronchial perspective. All drawings were superimposed over each other with reduced opacity. The average center point for all drawings in each lymph node position was marked with a star. The area marked with a line was covered by more than 3 of the 14 most experienced physicians (more than 1000 EBUS-TBNA performed). This area is presented as the new expert opinion map. It was not possible to present an expert opinion for 10R.

## Discussion

This study provided an expert opinion map of the mediastinal lymph nodes from an intrabronchial view for position 4 L, 4R, 7, 10 L, 11R and 11 L. The agreement was high among the most experienced physician for position 4 L, 7, 10 L, 11R and 11 L, in 4R the agreement was low. No area was covered by more than 20 % of the most experiences physicians in 10R.

The main strength of this study was the experience of the physicians. The 14 most experienced physicians have altogether performed more than 14000 EBUS-TBNA. When EBUS-TBNA is performed, EBUS gives the physician a clear picture of the lymph node. Simultaneously the bronchoscopic picture is available to show where the lymph node is localized. When hundreds of procedures have been performed, the operator should be able to form a sound opinion of the normal variation between patients. It was important to get the opinion of the most experienced physicians in this study; and 50 % of the physicians had performed more than 500 EBUS-TBNA procedures. 30 % of the physicians had performed more than 1000 EBUS-TBNA procedures, which represents a unique knowledge of the TBNA positions.

The main weakness of this study is the lack of patient measurements. The study reflects the expert opinion; no real life anatomical analyses were performed. Recall of previous maps and previous teaching of positions might bias the expert opinion. Another weakness in this study is the possibility to perform EBUS-TBNA based on the ultrasound landmarks with little or almost no concern about the intrabronchial landmarks [14]. The lack of standardized tools to define agreement among the experts was a challenge in the study.

The expert opinion map was validated by an interobserver analysis. The agreement of 14 experts who have performed more than 1000 EBUS represents a great repository of knowledge. The agreement was defined based on center point variation and area variation. The close relation between the variation of center points and variation of areas supports the validity of the analysis.

It was possible to draw a new expert opinion map for lymph node 4 L, 7, 10 L, 11R and 11 L with high agreement among the 14 most experienced physicians. The agreement among the most experienced physicians was significantly higher than the less experienced physicians in 10 L (p:0.01). There is a need to further validate the suggestion of optimal sampling from 4R because of low agreement. It was not possible to suggest an expert opinion for 10R.

The reason for the lack of agreement in 4R is not clear. The large possible area for sampling and the distance to the anatomical landmarks might explain some of the lack of agreement in station 4R. 4R extends from the caudal border of the innominate vein to the lower border of the azygos and includes the pretracheal nodes to the left lateral border of trachea [8]. The close relation to vena Azygos might change the physicians’ focus to the ultrasound picture. After station 7, 4R is the most punctured lymph node station [15]. The agreement among the most experienced and the others were lower than expected for 4R. 10R is more rarely punctured. The position is complicated just distal to the Azygos vein. It is often complicated to bend the EBUS-bronchoscope enough to get an optimal position for 10R. This might explain the lack of agreement.

## Conclusion

The usefulness of this map will depend on the approach to lymph nodes in mediastinum. Those who still use conventional TBNA have to rely on the knowledge of the intrabronchial sample positions in addition to the CT scans. EBUS-TBNA of one ore two enlarged lymph nodes (“hit and run”) can probably be performed without much attention to the intrabronchial landmarks, but for mapping of all lymph nodes in the mediastinum the knowledge of the anatomy is crucial for the performance [5]. When all lymph nodes in the mediastinum shall be evaluated, the approximately positions from inside the bronchial tree must be known, not only the position outside the bronchial tree provided in previous maps. The map in this study might help the physician to locate the lymph nodes and decrease the duration of the procedure. Further validation of the 4R position is necessary; it was not possible to make a suggestion for 10R.

## Abbreviations

NSCLC: Non-small cell lung cancer
TBNA: Transbronchial needle aspiration
EBUS: Endobronchial ultrasound
CT: Computer tomography
EBUS-TBNA: Endobronchial ultrasound guided transbronchial needle aspiration
ATS: American Thoracic Society
IASLC: The International Association for the Study of Lung Cancer
